# FBXO7 triggers caspase 8-mediated proteolysis of the transcription factor FOXO4 and exacerbates neuronal cytotoxicity

**DOI:** 10.1016/j.jbc.2021.101426

**Published:** 2021-11-17

**Authors:** Su Hyoun Lee, Sungyeon Jung, Yun Ju Lee, Minju Hyun, Kwang Chul Chung

**Affiliations:** Department of Systems Biology, College of Life Science and Biotechnology, Yonsei University, Seoul, South Korea

**Keywords:** Parkinson’s disease, FBXO7, FOXO4, caspase 8, 6-OHDA, neuronal cell death, AD, Alzheimer's disease, DISC, death-inducing signaling complex, DMEM, Dulbecco’s modified Eagle’s medium, ECL, enhanced chemiluminescence, FADD, FAS-associated death domain, FBS, fetal bovine serum, FBXO7, F-box only protein 7, HEK293, human embryonic kidney 293, HRP, horseradish peroxidase, MPP+, 1-methyl-4-phenylpyridium ion, NDDs, neurodegenerative diseases, 6-OHDA, 6-hydroxydopamine, PD, Parkinson’s disease, PEI, polyethylenimine, PRR, proline-rich region, ROS, reactive oxygen species, SCF, SKP1-Cullin1-F-box, TRAIL, TNF-related apoptosis-inducing ligand, Ubl, ubiquitin-like, UPS, ubiquitin-proteasome system, X-IAP, X-linked IAP

## Abstract

Parkinson’s disease (PD) is characterized by the progressive loss of midbrain dopamine neurons in the substantia nigra. Mutations in the F-box only protein 7 gene (*Fbxo7*) have been reported to cause an autosomal recessive form of early-onset familial PD. FBXO7 is a part of the SKP1-Cullin1-F-box (SCF) E3 ubiquitin ligase complex, which mediates ubiquitination of numerous substrates. FBXO7 also regulates mitophagy, cell growth, and proteasome activity. A member of the FOXO family, the transcription factor FOXO4, is also known to modulate several cellular responses, including cell cycle progression and apoptosis; however, the relationship between FBXO7 and FOXO4 has not been investigated. In this study, we determined that FBXO7 binds to FOXO4 and negatively regulates intracellular FOXO4 levels. Interestingly, we also found that FBXO7-mediated degradation of FOXO4 did not occur through either of two major proteolysis systems, the ubiquitin-proteasome system or the lysosome-autophagy pathway, although it was blocked by a caspase 8-specific inhibitor and *caspase 8*-knockdown. Moreover, intracellular FOXO4 levels were greatly reduced in dopaminergic MN9D cells following treatment with neurotoxic 6-hydroxydopamine (6-OHDA), which was produced upon FBXO7-mediated and caspase 8-mediated proteolysis. Taken together, these results suggest that FOXO4 is negatively regulated in FBXO7-linked PD through caspase 8 activation, suppressing the cytoprotective effect of FOXO4 during 6-OHDA-induced neuronal cell death.

Parkinson’s disease (PD) is a progressive neurodegenerative disorder that affects 1% of the population over the age of 60 years (1). A common neuropathological hallmark of PD is the progressive loss of dopamine neurons in the substantia nigra, as well as the formation of Lewy bodies, which are aggregates of misfolded α-synuclein protein. Mutations in several genes have been identified as the cause of familial forms of PD, including α-*synuclein*, *parkin*, *PINK1*, *LRRK2*, *DJ*-*1*, *FBXO7*, and *ATP13A2* (2, 3). Among these, mutations in *F*-*box only protein 7* (*FBXO7*) cause autosomal-recessive early-onset Parkinsonism with atypical features, including dementia, dystonia, hyperreflexia, and pyramidal signs (4).

FBXO7 is a member of the F-box protein family and is involved in both canonical and noncanonical functions. FBXO7 contains multiple domains, such as the N-terminal ubiquitin-like (Ubl) domain, signature F-box domain, and C-terminal unstructured proline-rich region. Canonically, FBXO7 functions as a part of the Skp1-Cullin1-F-box (SCF) E3 ubiquitin ligase complex and mediates the substrate-recognition adapter protein (5, 6). FBXO7 also functions to modulate protein stability of several substrates (7, 8, 9, 10, 11). In SCF-independent noncanonical roles, FBXO7 regulates cell cycle progression, intracellular trafficking, proteasome activity, and mitophagy (6). Specifically, FBXO7 interacts with Cdk6 and controls the formation of the cyclin D/Cdk6 complex (12, 13). Moreover, FBXO7 interacts with parkin *via* its Ubl domain to help recruit parkin to damaged mitochondria and initiate mitophagy (14). Interestingly, the PD-associated T22M mutation of the FBXO7 Ubl domain appears to impair its interaction with parkin.

The FOXO family is a subclass of forkhead transcription factors, including four mammalian members. FOXOs are involved in a number of physiological and pathological processes, including cell growth, differentiation, cell death, and aging (15). FOXO4 also plays a key role in various cellular processes, including cell growth, apoptosis, longevity, and oxidative stress signaling (16). For example, FOXO4 promotes cell cycle arrest by regulating the expression of antiproliferative genes in each phase, such as p27kip1, cyclin D1, and GADD45 (17, 18). Recent studies have revealed that FOXO factors act as crucial regulators in the nervous system. For instance, FOXOs play a role in triggering either neuronal survival or apoptosis in response to stress and are likely to be relevant in nervous system pathologies (19). In dopaminergic neurons overexpressing α-synuclein, FOXO3 induces the clearance of soluble α-synuclein and regulates neuronal cell death (20), while FOXO1 expression is increased in the frontal cortex of PD patients (21).

Caspase is a family of aspartate-specific cysteine proteases that plays an essential role in apoptotic cell death and inflammation. The caspase family members are classified by their known roles (22), including those involved in cell death as apoptotic initiators or apoptotic executioners and those involved in inflammatory responses. Among apoptotic initiators, caspase 8 plays a role in extrinsic apoptosis by combining with FAS-associated death domain (FADD) to form the death-inducing signaling complex (DISC). Moreover, active caspase 8 is released from the DISC, which sequentially activates the apoptotic effectors, such as caspases 7 and 3. Therefore, caspase 8 is able to promote apoptosis by either directly or indirectly activating downstream effector caspases (23).

FOXOs are involved in the aging process of nervous system, and their altered regulation or activity can be associated with age-related neurodegenerative diseases (NDDs), including Alzheimer’s disease (AD) and PD (19). However, little is known about this aspect of their activity. Furthermore, unlike FOXO1 and FOXO3, the neuronal function of FOXO4 is poorly understood. In the current study, we found that PD-associated FBXO7 interacted with FOXO4 in dopaminergic neuroblastoma MN9D cells, negatively regulating the stability of FOXO4 through the novel caspase 8-linked pathway. In addition, FOXO4 activity was reduced during 6-hydroxydopamine (6-OHDA)-induced apoptotic death in neuronal cells, which resulted from FBXO7-mediated proteolysis. Taken together, our findings suggest that the functional relationship between FBXO7 and FOXO4 may play a role in 6-OHDA-induced neuronal cell death, as well as PD pathogenesis.

## Results

### FBXO7 interacted with FOXO4 in mammalian cells

**Figure 1:**
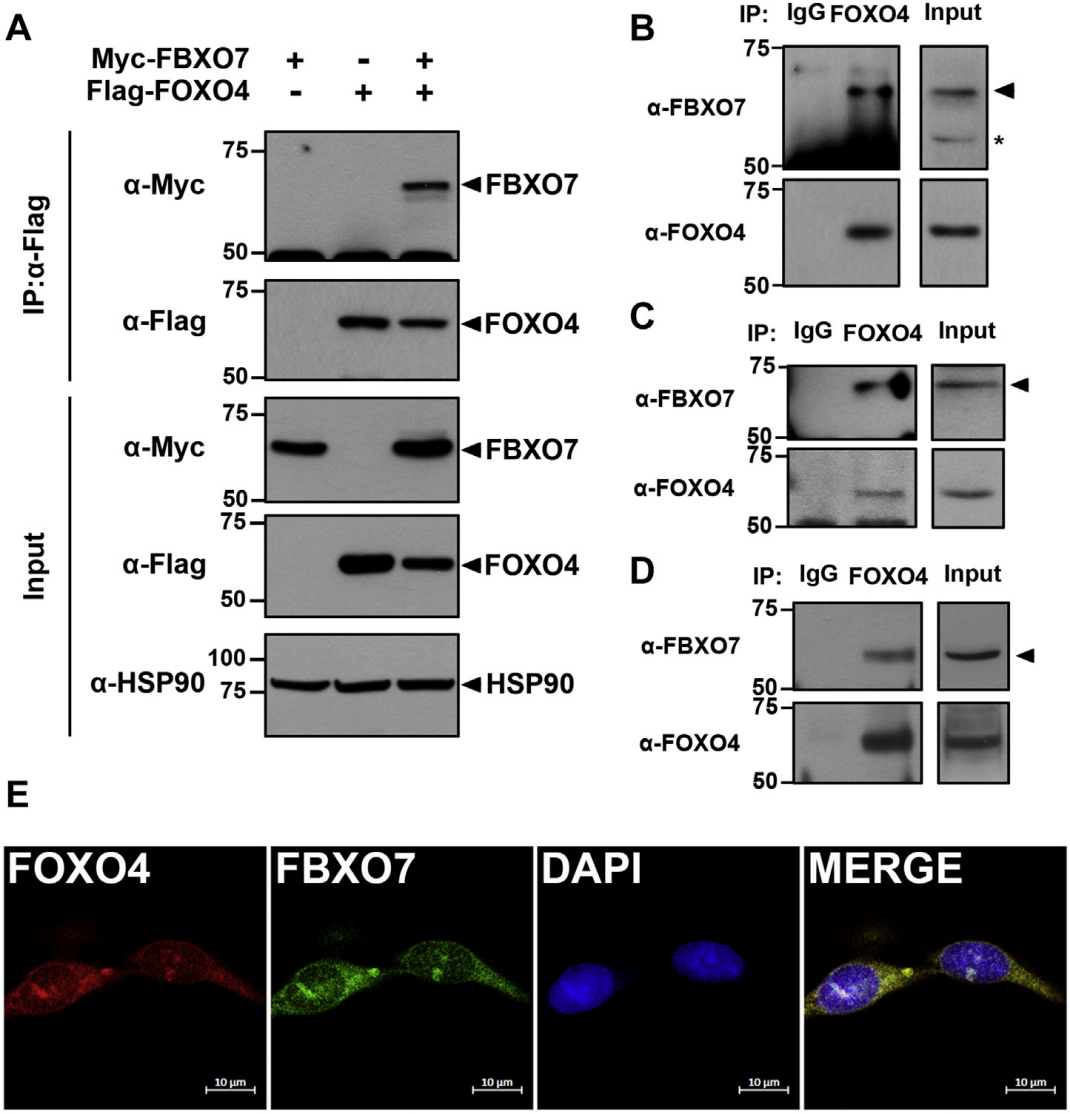
**FBXO7 interacts with FOXO4.***A*, HEK293 cell were transfected for 24 h with plasmids encoding Myc-FBXO7 and/or FLAG-FOXO4. Cell lysates were immunoprecipitated using an anti-FLAG antibody and the precipitates immunoblotted with the indicated antibodies. HSP90 served as a loading control. *B*, HEK293 cell-lysates were immunoprecipitated using either an anti-FOXO4 antibody or preimmune IgG (control), followed by immunoblotting with the indicated antibodies. *C*, MN9D cell lysates were immunoprecipitated using an anti-FOXO4 IgG, followed by immunoblotting with the indicated antibodies. *D*, mouse brain lysates were immunoprecipitated using anti-FOXO4 IgG, followed by immunoblotting with the indicated antibodies. *E*, SH-SY5Y cells were fixed, permeabilized, and immunostained. Representative confocal images of cells immunostained for endogenous FOXO4 (*red*) and endogenous FBXO7 (*green*) are shown. Nuclei were counterstained with DAPI (*blue*). Scale bars denote 10 μm.

It has been recently reported that defective FOXO1 and FOXO3 activities are observed in both a PD mouse model and patients with PD (19). However, the putative role of FOXO4 in PD pathology and its underlying mechanism are poorly understood. In the current study, we investigated whether and how FOXO4 was biochemically and functionally linked to the PD-linked gene product FBXO7. We first examined whether FBXO7 binds to FOXO4 in mammalian cells. HEK293 cells were transfected with plasmids encoding Myc-tagged FBXO7, either alone or together with FLAG-FOXO4. Immunoprecipitation of cell lysates was then performed using anti-FLAG antibody. Immunoblotting of the anti-FLAG immunocomplexes with anti-Myc antibody revealed that the ectopically expressed FBXO7 interacted with FOXO4 in HEK293 cells (Fig. 1*A*). In addition, the interaction between endogenous FBXO7 and endogenous FOXO4 was confirmed in HEK293 cells, mouse neuroblastoma MN9D cells, and whole mouse brain lysates (Fig. 1, *B*–*D*). Immunohistochemical analysis of human neuroblastoma SH-SY5Y cells revealed that endogenous FBXO7 and FOXO4 colocalized in cells (Fig. 1*E*).

To determine the FBXO7 domain(s) responsible for FOXO4 binding, several FLAG-tagged FBXO7-deletion mutants were constructed (Fig. S1*A*). The mutants included variants lacking the N-terminal Ubl domain (ΔU), F-box (ΔF), C-terminal region spanning amino acids 376–522 (ΔC), or N-terminal region of amino acid 1–333 (ΔN). HEK293 cells were transfected with plasmids encoding either FLAG-tagged wild-type FBXO7 or one of the deletion mutants, and coimmunoprecipitation analysis was performed using an anti-FOXO4 antibody. Immunoblotting of the samples with anti-FOXO4 antibody revealed that FOXO4 is bound to FBXO7-ΔF and FBXO7-ΔC, as well as full-length FBXO7. However, this interaction was not observed with the FBXO7-ΔU or FBXO7-ΔN fragments, which both lacked the N-terminal Ubl domain (Fig. S1*B*). Taken together, these results suggested that FOXO4 binds to FBXO7 in mammalian cells and that the Ubl domain of FBXO7 is required for this interaction.

### FBXO7 decreased FOXO4 protein stability

**Figure 2:**
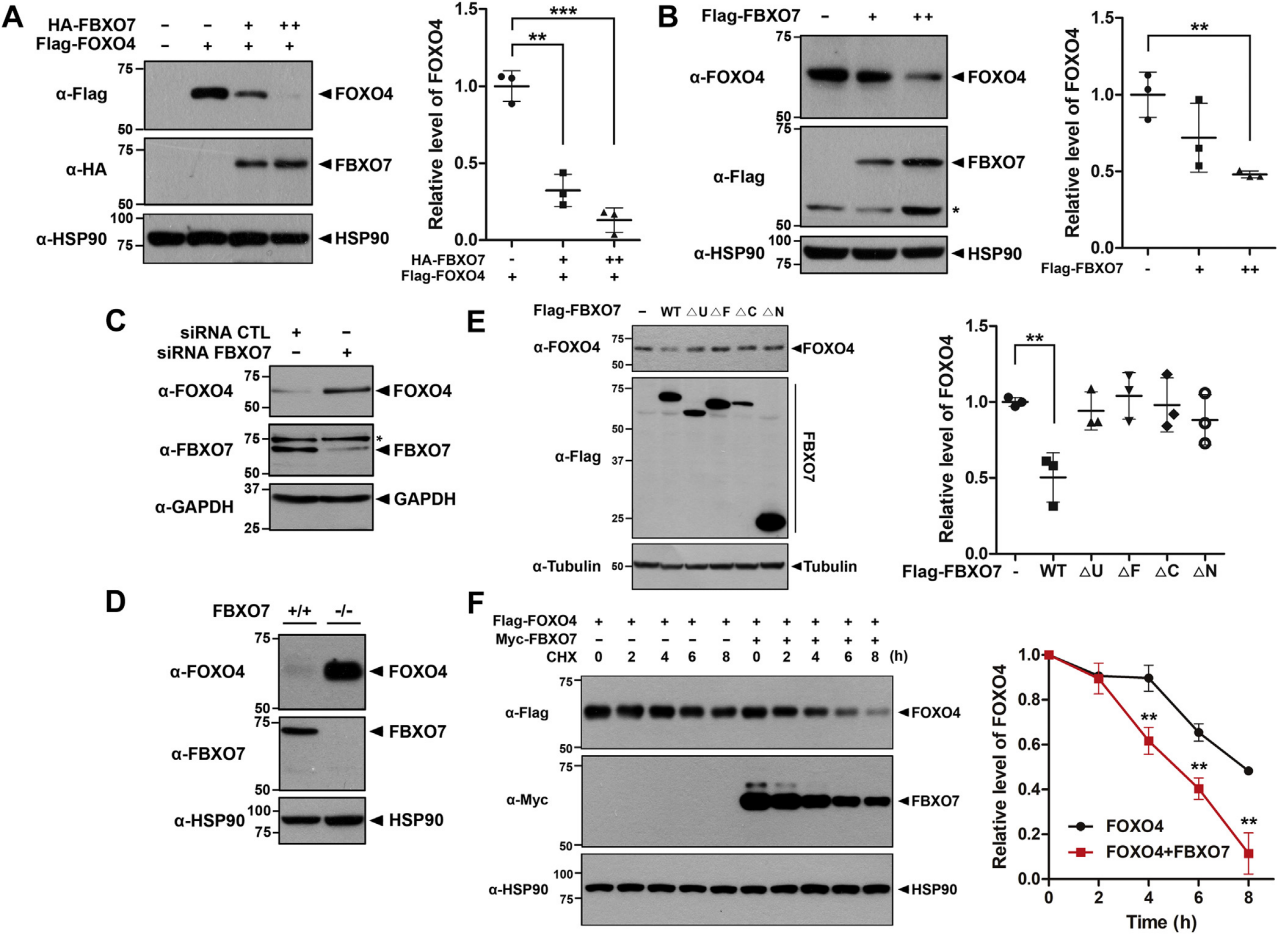
**FBXO7 decreases FOXO4 protein stability.***A*, HEK293 cells were transfected for 24 h with a plasmid encoding FLAG-FOXO4, either alone or in combination with increasing amounts of a plasmid encoding HA-FBXO7. Transfected cell lysates were immunoblotted and screened with anti-FLAG or anti-HA antibodies. Relative levels of FOXO4 were quantified and the results presented as the mean ± SD of three independent experiments (∗∗∗*p* ≤ 0.0001; ∗∗*p* ≤ 0.001). *B*, HEK293 cells were transfected for 24 h with increasing amounts of plasmid encoding FLAG-FBXO7. Transfected cell lysates were immunoblotted and screened with anti-FOXO4 or anti-FLAG antibodies. Relative levels of FOXO4 were quantified and results presented as the mean ± SD of three independent experiments (∗∗*p* ≤ 0.001). *C*, HEK293 cells were transfected for 48 h with siRNA-control (CTL) or siRNA-*FBXO7*. Transfected cell lysates were immunoblotted and screened with anti-FOXO4 or anti-FBXO7 antibodies. *D*, Immunoblotting analysis of lysates of FBXO7^+/+^ and FBXO7^−/−^ HAP1 cells was performed using anti-FOXO4 or anti-FBXO7 antibodies. *E*, HEK293 cells were transfected for 24 h with a plasmid encoding FLAG-tagged wild-type FBXO7 or its deletion mutants. Transfected cell lysates were immunoblotted and screened with anti-FOXO4 or anti-Flag antibodies. Relative levels of FOXO4 were quantified and the results presented as the mean ± SD of three independent experiments (∗∗*p* ≤ 0.001). *F*, HEK293 cells were transfected for 24 h with plasmids encoding FLAG-FOXO4 and/or Myc-FBXO7. Cells were treated for the indicated times with 25 μg/ml cycloheximide, and cell lysates were immunoblotted with the indicated antibodies. Relative levels of FOXO4 were quantified and the results presented as the mean ± SD of three independent experiments (∗∗*p* ≤ 0.001). Hsp90, GAPDH, and Tubulin served as loading controls.

To gain further insight into the link between FBXO7 and FOXO4, we investigated whether FBXO7, as a component of ubiquitin E3 ligase, may affect the level of FOXO4. As shown in Figures 1*A* and 2, *A* and *B*, exogenous and endogenous levels of FOXO4 were greatly reduced in the presence of FBXO7 in a dose-dependent manner. In contrast, intracellular levels of FBXO7 were unaffected, irrespective of FOXO4 (Fig. 2*A*). Similar to the patterns shown in Figure 2, *A* and *B*, endogenous FOXO4 levels were significantly increased in HEK293 cells by siRNA-mediated *FBXO7* knockdown (Fig. 2*C*), suggesting that FBXO7 negatively regulated FOXO4 activity. This was further supported by the finding that endogenous FOXO4 levels were significantly increased in *FBXO7*-knockout HAP1 cells (Fig. 2*D*).

**Figure 3:**
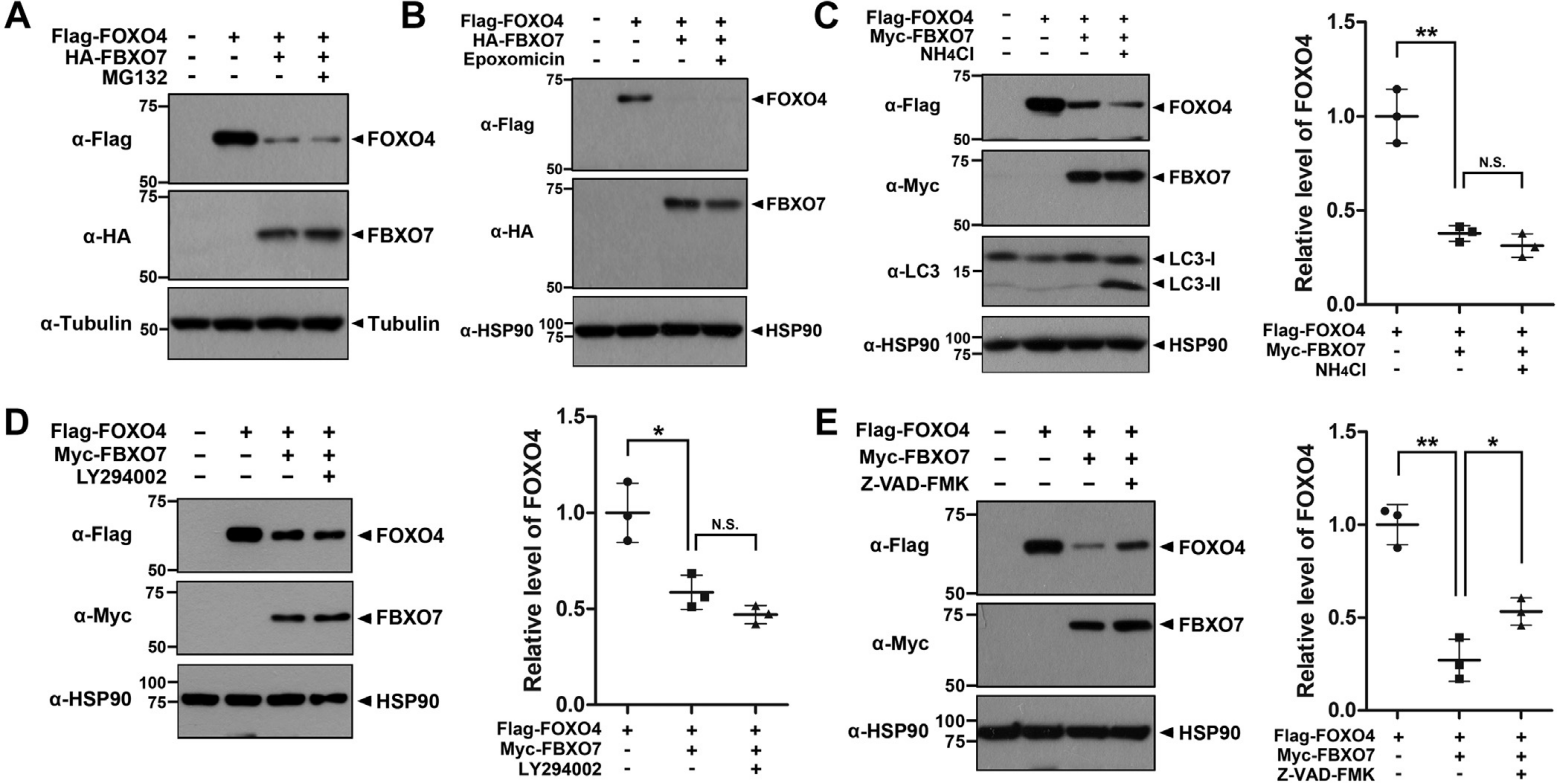
**FBXO7 decreases FOXO4 stability through caspase activation.***A* and *B*, HEK293 cells were transfected for 24 h with plasmids encoding FLAG-FOXO4 and/or HA-FBXO7. Cells were then treated for an additional 6 h with vehicle (−), 10 μM MG132 (*A*), or 1 μM epoxomicin (*B*). *C*, HEK293 cells were transfected for 24 h with plasmids encoding FLAG-FOXO4 and/or Myc-FBXO7. Cells were then treated for additional 6 h with vehicle (−) or 25 mM NH_4_Cl. Relative levels of FOXO4 were quantified and the results presented as the mean ± SD of three independent experiments (∗∗*p* ≤ 0.001; N.S., not significant). *D*, HEK293 cells were transfected for 24 h with plasmids encoding FLAG-FOXO4 and/or Myc-FBXO7. Cells were then treated for additional 4 h with vehicle (−) or 50 μM LY294002. Relative levels of FOXO4 were quantified and the results presented as the mean ± SD of three independent experiments (∗*p* ≤ 0.05; N.S., not significant). *E*, HEK293 cells were transfected for 24 h with plasmids encoding FLAG-FOXO4 and/or Myc-FBXO7. The cells were then treated for additional 6 h with vehicle (−) or 100 μM Z-VAD-FMK. *H*, relative levels of FOXO4 were quantified and the results presented as the mean ± SD of three independent experiments (∗∗*p* ≤ 0.001; ∗*p* ≤ 0.05). Tubulin and Hsp90 served as a loading control.

To investigate which protein domain of FBXO7 plays a role in the regulation of FOXO4 level, HEK293 cells were transfected with plasmids encoding either FLAG-FBXO7-WT or one of its deletion mutants. Immunoblotting analysis of cell lysates using anti-FOXO4 antibodies revealed that, except the FBXO7-WT, the FOXO4 level was unaffected by all four deletion mutants (Fig. 2*E*). These results suggested that not the discrete domain within the FBXO7 protein but its whole structure appears to be required for the catalytic activity of FBXO7 and FBXO7-mediated downregulation of FOXO4.

To determine whether FBXO7 regulated the protein stability of FOXO4, HEK293 cells were transfected with plasmids encoding FLAG-tagged FOXO4, either alone or in combination with Myc-FBXO7. The cells were then treated with 25 μM cycloheximide for the indicated times, and immunoblot analysis of the cell lysates was performed using an anti-FLAG antibody. The immunoblotting revealed that the half-life of FOXO4 was markedly reduced by FBXO7 (Fig. 2*F*). However, this effect was not seen with FBXO7-ΔU (Fig. S2). These results suggest that FBXO7-ΔU cannot mediate the proteolysis of FOXO4, possibly due to the lack of their binding.

Collectively, the overall results suggested that FBXO7 promoted the degradation of FOXO4.

### FBXO7 decreased FOXO4 stability through caspase activation

Two major types of intracellular protein degradation machinery exist in eukaryotic cells, the ubiquitin-proteasome system (UPS) and lysosome-mediated autophagy pathway (24). Therefore, we next determined which pathway operates in FBXO7-mediated proteolysis of FOXO4. HEK293 cells were transfected for 24 h with plasmids encoding FLAG-FOXO4, HA-FBXO7, or Myc-FBXO7, either alone or in combination, and treated the transfected cells with either proteasome inhibitor (MG132 or epoxomicin) or lysosomal autophagy inhibitor (NH_4_Cl or LY294002). As shown in Figure 3, *A* and *B*, pretreatment with proteasome inhibitors had no effect on FBXO7-mediated reduction of FOXO4 levels. These results indicated that the reduction of FOXO4 induced by FBXO7 occurred through SCF-independent and UPS-independent pathways. In addition, lysosomal inhibitors did not affect the proteolytic action of FBXO7 on FOXO4, suggesting that the proteolysis did not occur through the lysosomal autophagy pathway (Fig. 3, *C* and *D*).

We then examined whether FBXO7 promoted the reduction of FOXO4 through a caspase-dependent pathway. To investigate this possibility, HEK293 cells were transfected with plasmids encoding FLAG-FOXO4 and Myc-FBXO7, either alone or in combination, and the transfected cells were treated with the pan-caspase inhibitor Z-VAD-FMK. As shown in Figure 3*E*, immunoblotting analysis of cell lysates using anti-FLAG antibodies revealed that the presence of a caspase inhibitor markedly increased the reduction of FOXO4 levels. These results indicated that the FBXO7-mediated proteolysis of FOXO4 occurred through caspase activation. However, the pretreatment of caspase inhibitor could not recover the FOXO4 level completely by FBXO7, and so presumably, the caspase-independent mechanisms may also be involved in the reduction of FOXO4 level.

### FBXO7 promoted FOXO4 degradation *via* caspase 8 activation

**Figure 4:**
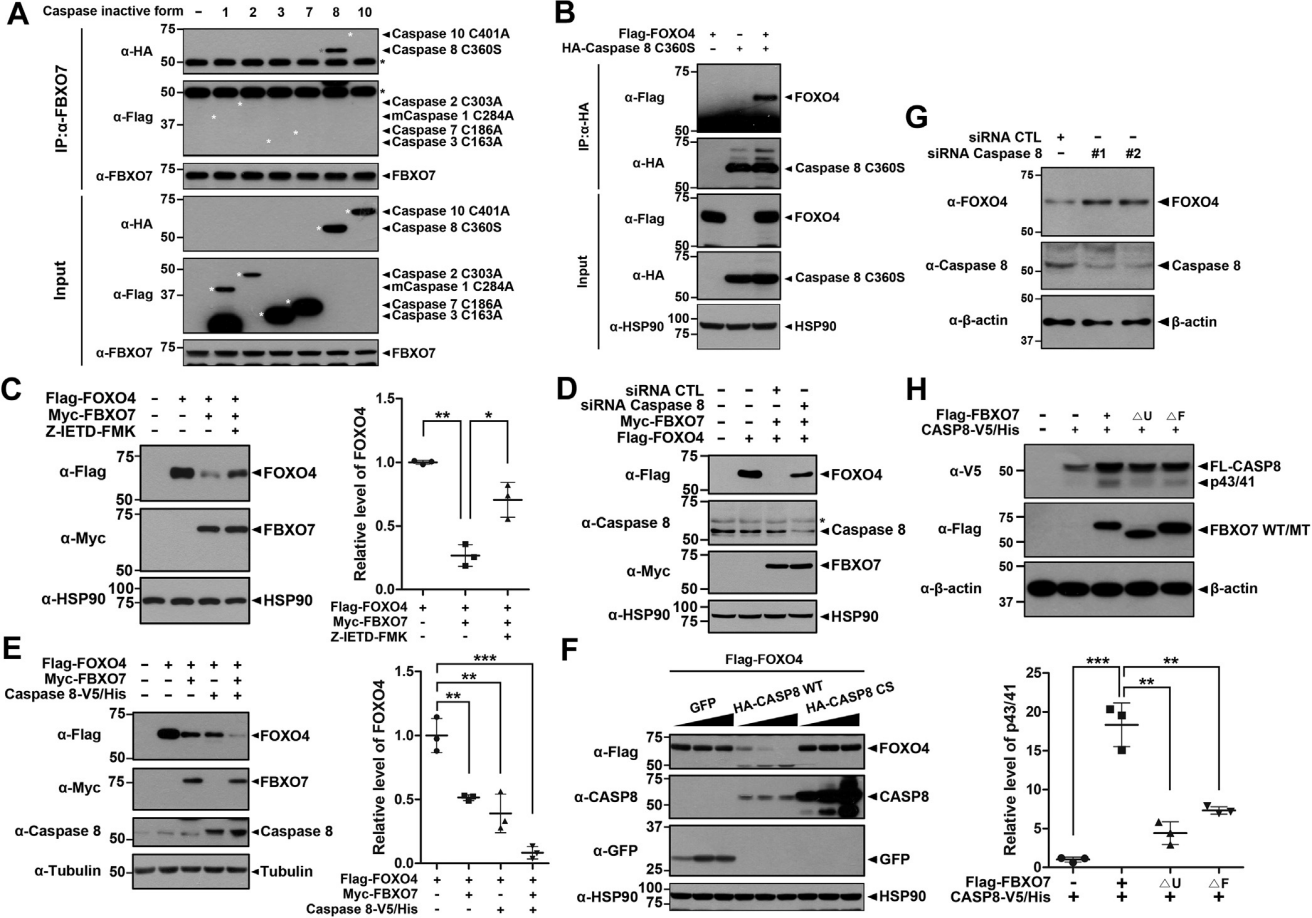
**FBXO7 decreases FOXO4 stability through a caspase 8 activation.***A*, HEK293 cells were transfected for 24 h with the plasmids encoding FLAG-caspase 1-C284A, FLAG-caspase 2-C303A, FLAG-caspase 3-C163A, FLAG-caspase 7-C186A, HA-caspase 8-C360S, or HA-caspase 10-C401A. Cell lysates were immunoprecipitated using anti-FBXO7 antibody followed by immunoblotting with the indicated antibodies. *B*, HEK293 cells were transfected for 24 h with plasmids encoding HA-caspase 8-C360S and/or FLAG-FOXO4. Cell lysates were immunoprecipitated with anti-HA antibody. The precipitates were immunoblotted screened using the indicated antibodies. *C*, HEK293 cells were transfected for 24 h with plasmids encoding FLAG-FOXO4 and/or Myc-FBXO7. The cells were then treated for additional 3 h with vehicle (−) or Z-IETD-FMK (20 μM). Relative levels of FOXO4 were quantified and the results presented as the mean ± SD of three independent experiments (∗∗*p* ≤ 0.001; ∗, *p* ≤ 0.05). *D*, HEK293 cells were transfected for 48 h with siRNA-control (CTL), siRNA-*caspase 8*, a plasmid encoding FLAG-FOXO4, or a plasmid encoding Myc-FBXO7, either alone or in combination. Cell lysates were immunoblotted and screened using the indicated antibodies. *E*, HEK293 cells were transfected for 24 h with plasmids encoding FLAG-FOXO4, Myc-FBXO7, or caspase 8-V5/His, either alone or in combination. Cell lysates were immunoblotted and screened using the indicated antibodies. Relative levels of FOXO4 were quantified and the results presented as the mean ± SD of three independent experiments (∗∗∗*p* ≤ 0.0001; ∗∗*p* ≤ 0.001). *F*, HEK293 cells were transfected for 24 h with a plasmid encoding FLAG-FOXO4, either alone or in combination with increasing amounts of a plasmid encoding HA-tagged wild-type caspase 8 (CASP8) or its C360S mutant. Transfected cell lysates were immunoblotted and screened using the indicated antibodies. *G*, HEK293 cells were transfected for 48 h with control siRNA (CTL), *caspase 8*-siRNA #1 or #2. Transfected cell lysates were immunoblotted and screened with anti-FOXO4 or anti-caspase 8 antibodies. *H*, HEK293 cells were transfected for 24 h with a plasmid encoding caspase 8-V5/His, Flag-FBXO7-WT, Flag-FBXO7-ΔU, or Flag-FBXO7-ΔF alone or in combination. Cell lysates were immunoblotted and screened using the indicated antibodies. Relative levels of p43/41 were quantified and the results presented as the mean ± SD of three independent experiments (∗∗∗, *p* ≤ 0.0001; ∗∗, *p* ≤ 0.001). Hsp90, β-actin, and Tubulin served as loading controls.

We further examined how the caspase pathway was linked downstream of FBXO7 and its underlying mechanism. For example, it was of interest which caspase(s) functionally interacted with FBXO7. Cells were transfected with plasmids encoding FLAG-tagged catalytic-inactive caspases 1, 2, 3, and 7 or HA-tagged catalytically inactive caspase 8 and 10. Cell lysates were immunoprecipitated using anti-FLAG or anti-HA antibodies, accordingly. The immunoprecipitated samples were then immunoblotted using anti-FBXO7 antiserum, which revealed that exogenously added catalytic-inactive caspase 8 interacted with FBXO7 in HEK293 cells (Fig. 4*A*). However, this binding was not observed with caspases 1, 2, 3, 7, or 10 (Fig. 4*A*).

To determine which region of FBXO7 is responsible for its interaction with caspase 8, HEK293 cells were transfected with caspase 8-V5/His alone together with FBXO7-WT or one of its deletion mutants, and coimmunoprecipitation was performed. As shown in Figure S3*B*, immunoblot analysis of anti-FLAG immune complexes with anti-V5 IgG revealed that caspase 8 binds to FBXO7-ΔF and FBXO7-ΔC, as well as FBXO7-WT. However, this interaction was not seen with FBXO7-ΔU or FBXO7-ΔN mutant. These results suggest that caspase 8 binds to the FBXO7 *via* its N-terminal Ubl domain.

To verify the biochemical interaction between FOXO4 and caspase 8, HEK293 cells were transfected with plasmid encoding the HA-tagged catalytically inactive caspase 8-C360S mutant and FLAG-FOXO4, either alone or in combination. Coimmunoprecipitation assays revealed that ectopically expressed FOXO4 is bound to caspase 8 (Fig. 4*B*).

To determine whether caspase 8 was involved in the FBXO7-induced degradation of FOXO4, we evaluated the effect of the caspase 8-specific inhibitor Z-IETD-FMK. Similar to the effects observed for Z-VAD-FMK, pretreatment with Z-IETD-FMK considerably inhibited the FBXO7 proteolysis of FOXO4 (Fig. 4*C*). Furthermore, siRNA-mediated knockdown of *caspase 8* expression blocked the degradation of FOXO4 (Fig. 4*D*).

Conversely, the overexpression of caspase 8 plus FBXO7 enhanced FBXO7-mediated FOXO4 degradation compared with that of cells transfected with FBXO7 alone (Fig. 4*E*). However, this was not observed in cells transfected with catalytically inactive caspase 8-C360S (Fig. 4*F*). In addition, endogenous FOXO4 level was reversely increased by siRNA-mediated knockdown of caspase 8 expression (Fig. 4*G*). Taken together, these results indicated that the caspase 8 is involved in the FBXO7-mediated FOXO4 degradation. However, the FOXO4 level was not completely recovered by peptide inhibitor of caspase 8 or *caspase 8*-specific siRNA. So, we assume that the caspase 8-dependent pathway as well as caspase 8-independent novel mechanism may operate together in the downstream steps of FBXO7-induced FOXO4 proteolysis reaction.

Next, we examined whether FBXO7 activates the caspase 8. Upon activation, full-length caspase 8 is known to be processed into two auto-cleavage fragments having the sizes of 43/41 and 19 kDa (denoted as p43/p41 and p19, respectively) (25). Based on this report, cells were transfected with the plasmid encoding caspase 8-V5/His alone or together with one of FLAG-FBXO7-WT, FLAG-FBXO7-ΔU, or FLAG-FBXO7-ΔF, and we checked which cells could generate the cleavage intermediate of caspase 8, p43/p41. Immunoblotting analysis of cell lysates demonstrated that cells overexpressing exogenous FBXO7-WT produced a greater level of p43/41 form by more than 18-fold than mock-transfected control cells (Fig. 4*H*). However, this effect was not seen with cells transfected with FBXO7-ΔU and FBXO7-ΔF mutant (Fig. 4*H*). Taken together, these results suggested that FBXO7 activates the caspase 8.

### Caspase 8 is indirectly involved in FOXO4 degradation

Next, we tried to examine whether caspase 8 directly cleaves FOXO4, and if it occurs, to identify the cleavage site(s) of caspase 8 on the FOXO4. Caspases cleave their substrates almost exclusively after Asp residues (26). Several studies have suggested the preferred cleavage sequences of caspase 8 (Fig. S4*A*) (27, 28, 29, 30). Based on these sequences, nine point mutants of FOXO4 having the substitution at highly probable 9 Asp sites with Ala, respectively, out of total 23 Asp sites in human FOXO4 protein were generated (*i.e.*, D21A, D53A, D143A, D251A, D373A, D473A, D482A, D492A, and D504A; Fig. S4*B*). We then examined whether FBXO7 could still digest each of these mutants. Immunoblotting analysis revealed that caspase 8 cleaved all those nine point mutants of FOXO4 to a similar extent of wild-type FOXO4 (Fig. S4*C*). To further determine whether caspase 8 cleaves nonconserved site of FOXO4, the remaining 13 Asp sites (*i.e.*, D17 and 19A, D140A, D199A, D308A, D380A, D387A, D464A, D469 and 471A, D484A, D489A, D498A) that do not include the substrate cleavage sequence of caspase 8 were additionally mutated (27, 28, 29, 30), excluding the D2 residue since it is close to the amino terminus. As shown in Figure S4*D*, all those 13 point mutants of FOXO4 were still cleaved by caspase 8. These results indicated that FBXO7-mediated caspase 8 activation appeared to be indirectly involved in FOXO4 degradation, although the detailed working mechanism is not clarified, which could occur through additional unknown target(s).

### FBXO7 decreased FOXO4 transcriptional activity

**Figure 5:**
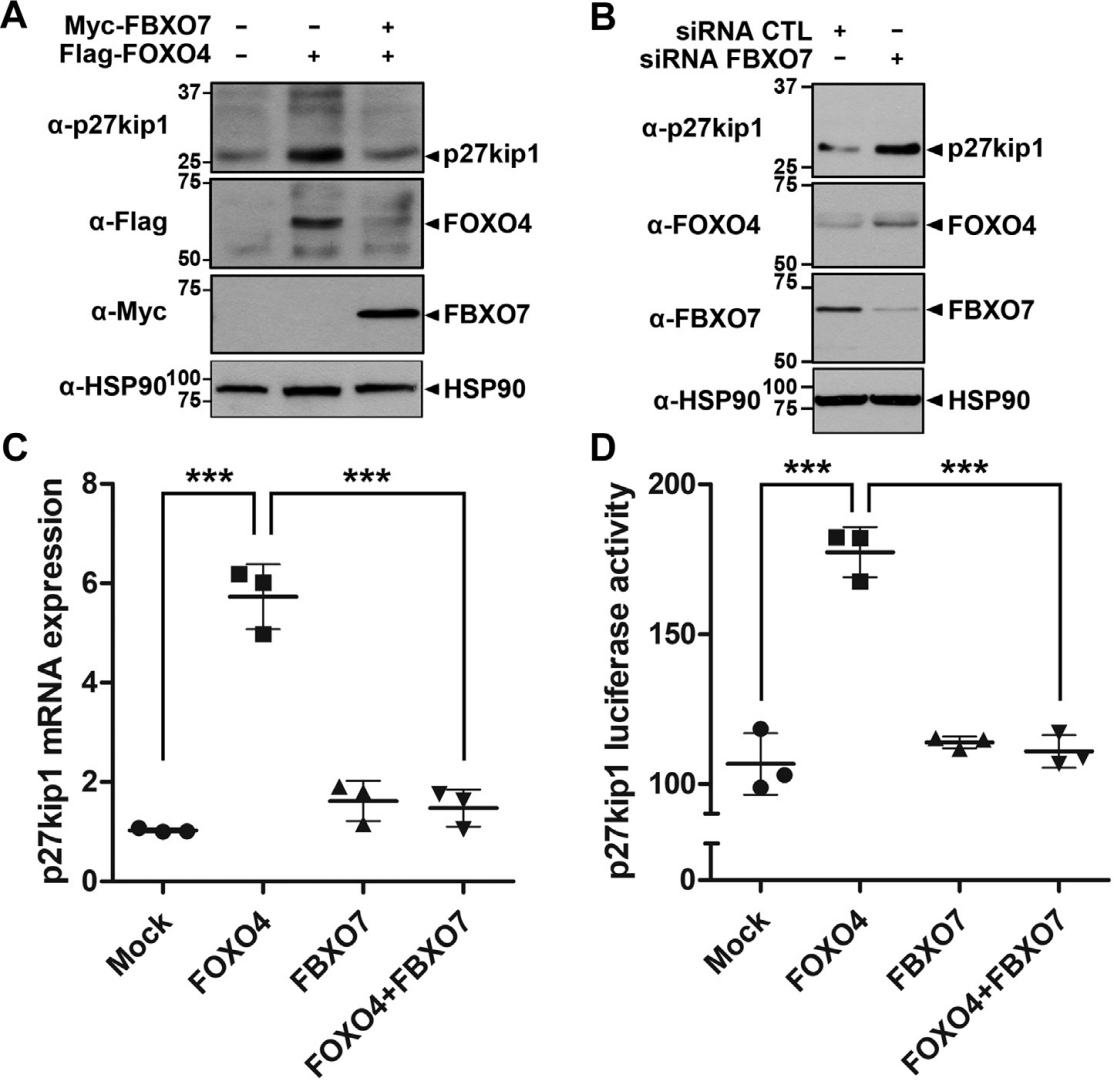
**FBXO7 decreases the transcription activity of FOXO4.***A*, HEK293 cells were transfected for 24 h with a plasmid encoding FLAG-FOXO4, either alone or in combination with a plasmid encoding Myc-FBXO7. Transfected cell lysates were immunoblotted and screened using anti-p27kip1, anti-FLAG, or anti-Myc antibody. *B*, HEK293 cells were transfected for 48 h with siRNA-control (CTL), or siRNA-*FBXO7*, and the cell lysates were immunoblotted and screened using anti-p27kip1, anti-FOXO4, or anti-FBXO7 antibody. Hsp90 served as a loading control. *C*, HEK293 cells were transfected for 24 h with plasmids encoding FLAG-FOXO4 and/or Myc-FBXO7. Total RNA was extracted and reverse transcribed. Levels of *p27kip1* mRNA were assessed using real-time PCR. Data are presented as the mean ± SD of three independent experiments (∗∗∗*p* ≤ 0.0001). *D*, MCF7 cells were cotransfected with the pGL3-p27kip1-Luc plasmid and the indicated plasmids. Following the incubation for 24 h, firefly luciferase activity was measured and normalized to the Renilla luciferase activity. Data are presented as the mean ± SD of three independent experiments (∗∗∗*p* ≤ 0.0001).

The transcription factor FOXO4 plays a role in cell cycle arrest and apoptosis (31). For instance, FOXO4 regulates transcription of cyclin-dependent kinase inhibitor p27kip1 (17). Based on that report, we determined whether FBXO7, through proteolysis of FOXO4, controlled the transcription of *p27kip1*, which is a typical downstream target gene of FOXO4. To initially examine whether FBXO7-meditaed degradation of FOXO4 regulated the endogenous levels of p27kip1 protein, HEK293 cells were transfected with plasmids encoding FLAG-FOXO4, either alone or in combination with Myc-FBXO7. Immunoblot analysis of cell lysates using an anti-p27kip1 antibody revealed that p27kip1 levels were significantly increased by the transfection of FOXO4 alone. In contrast, cotransfection of both FOXO4 and FBXO7 resulted in decreased p27kip1 levels *via* the reduced levels of FOXO4 (Fig. 5*A*). Meanwhile, knockdown of *FBXO7* expression using *FBXO7*-specific siRNA markedly increased p27kip1 levels (Fig. 5*B*).

We then assessed changes in *p27kip1* mRNA expression in the absence and presence of FBXO7. Real-time PCR analysis revealed that cells overexpressing exogenous FOXO4, which was used as a positive control, displayed 4.8-fold greater induction of *p27kip1* mRNA levels. There was no considerably change in *p27kip1* mRNA levels compared with that with the mock-transfected cells, regardless of FBXO7 (Fig. 5*C*). Moreover, cells coexpressing exogenous FOXO4 and FBXO7 exhibited significantly reduced *p27kip1* mRNA levels compared with that of cells overexpressing FOXO4 alone (Fig. 5*C*). We further measured the effect of FBXO7 on *p27kip1*-specific activity using a dual luciferase reporter assay. Consistent with the pattern of *p27kip1* mRNA expression determined by the PCR analysis, cells overexpressing exogenous FOXO4 showed 1.6-fold higher *p27kip1*-specific luciferase activity compared with that of mock-transfected control cells. In addition, cells coexpressing exogenous FOXO4 and FBXO7 exhibited greatly reduced *p27kip1* luciferase activity compared with that of cells transfected with FOXO4 alone (Fig. 5*D*). Taken together, these results suggested that FBXO7 negatively regulated the transcriptional activity of FOXO4 *via* its protein degradation.

### FBXO7 exacerbated 6-OHDA-induced neuronal cell death *via* negative regulation of FOXO4 levels

**Figure 6:**
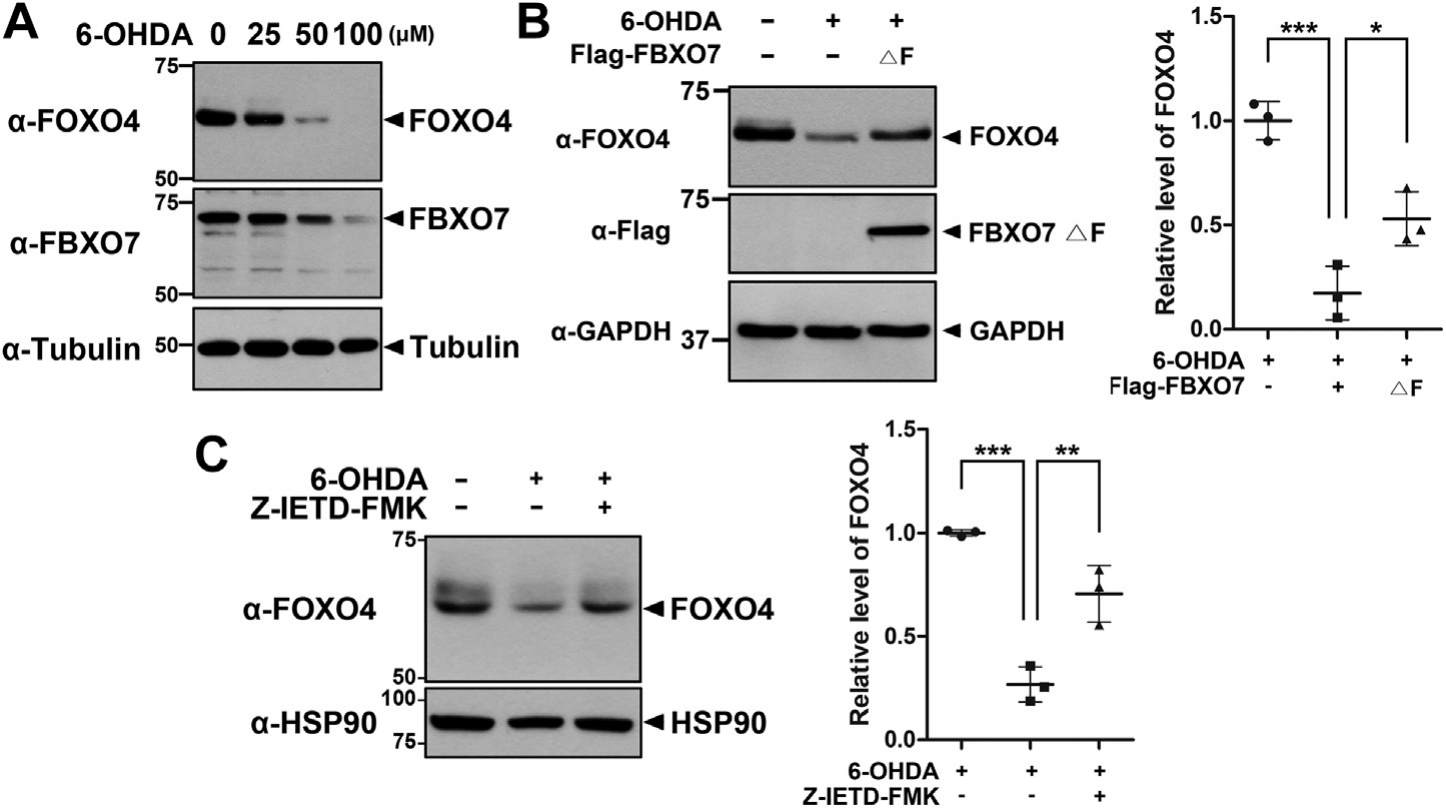
**Treatment with 6-OHDA reduces FOXO4 levels *via* FBXO7-mediated proteolysis.***A*, MN9D cells were treated for 6 h with vehicle (−) or 6-OHDA. Cell lysates were then immunoblotted with anti-FOXO4 or anti-FBXO7 antibodies. *B*, MN9D cells were transfected for 24 h with a plasmid encoding FLAG-FBXO7-ΔF mutant and then treated for additional 6 h with vehicle (−) or 6-OHDA (50 μM). Cell lysates were immunoblotted and screened using anti-FOXO4 or anti-FLAG antibodies. Relative levels of FOXO4 were quantified and the results presented as the mean ± SD of three independent experiments (∗∗∗*p* ≤ 0.0001; ∗*p* ≤ 0.05). *C*, MN9D cells were left untreated or treated for 3 h with 6-OHDA (50 μM), either alone or in combination with Z-IETD-FMK (20 μM). Cell lysates were immunoblotted and screened using anti-FOXO4 antibody. Relative levels of FOXO4 were quantified and the results presented as the mean ± SD of three independent experiments (∗∗∗*p* ≤ 0.0001; ∗∗*p* ≤ 0.001). Tubulin, GAPDH, and Hsp90 served as loading controls.

Treatment of neuronal cells with 6-OHDA produced oxidative stress through the generation of reactive oxygen species (ROS). The toxic effect of 6-OHDA in dopaminergic neurons reflects pathological circumstances and cell death in PD (32). In addition, several reports have revealed that 6-OHDA treatment triggers dysregulated autophagy in neuronal cells through caspase-dependent cell death (33). Based on these reports, the current finding of caspase-8 activation being involved in FBXO7-induced proteolysis of FOXO4 raises the possibility that this novel pathway may be activated or/and contribute to neuronal cell death in response to 6-OHDA. To evaluate the validity of this possibility, we tested the effect of 6-OHDA treatment on the proteolysis of FOXO4 in mouse dopaminergic MN9D cells. When the cells were exposed to 6-OHDA, endogenous FOXO4 levels were greatly reduced in a dose-dependent manner (Fig. 6*A*). Interestingly, FBXO7 levels were also reduced in a dose-dependent but in a less sensitive manner than that of FOXO4. To determine whether FBXO7 affected 6-OHDA-induced cell death and to identify the underlying mechanism, MN9D cells were transfected with plasmids encoding FLAG-FBXO7-ΔF, which lacked the F-box domain at the C-terminus. Cells treated with 6-OHDA and expressing the dominant-negative FLAG-FBXO7-ΔF mutant showed 3-fold higher levels of FOXO4 compared with cells treated with 6-OHDA alone (Fig. 6*B*). Based on a previous finding that 6-OHDA treatment mediates caspase-8 activation in MN9D cells (34), we examined whether Z-IETD-FMK could rescue 6-OHDA-mediated reduction of FOXO4 levels. Immunoblot analysis revealed that FOXO4 levels increased 2.6-fold in MN9D cells cotreated with 6-OHDA and Z-IETD-FMK compared with that in MN9D cells treated with 6-OHDA alone (Fig. 6*C*). These results suggested that 6-OHDA treatment caused FOXO4 proteolysis in MN9D cells through the action of both FBXO7 and caspase 8.

**Figure 7:**
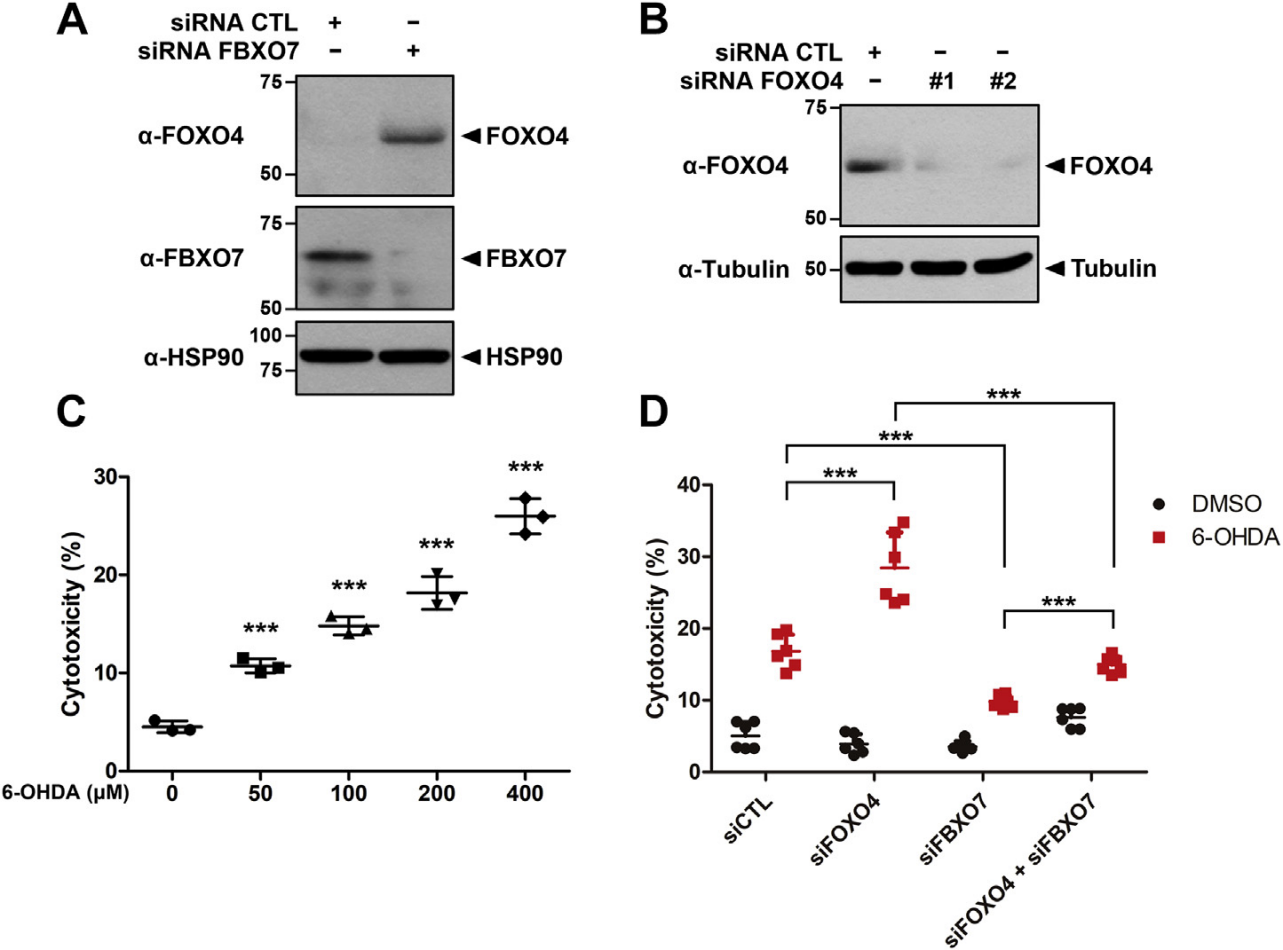
**FBXO7 reduces cytoprotective effects of FOXO4 through 6-OHDA-induced cell death.***A*, MN9D cells were transfected for 48 h with control siRNA (CTL) or *FBXO7*-siRNA, and the cell lysates were immunoblotted and screened using anti-FOXO4 or anti-FBXO7 antibodies. *B*, MN9D cells were transfected for 48 h with control siRNA (CTL), *FOXO4*-siRNA #1, or *FOXO4*-siRNA #2. Cell lysates were immunoblotted and screened using the indicated antibodies. Hsp90 and Tubulin served as loading controls. *C*, MN9D cells were treated for 4 h with DMSO (vehicle) or the indicated concentrations of 6-OHDA. Cell toxicity was measured using LDH assays. Data are presented as the mean ± SD of three independent experiments (∗∗∗*p* ≤ 0.0001). *D*, MN9D cells were transfected for 48 h with siRNA-control (CTL), siRNA-*FOXO4*, or siRNA-*FBXO7* and then treated for additional 4 h with DMSO or 6-OHDA (100 μM). Data are presented as the mean ± SD of six independent experiments (∗∗∗*p* ≤ 0.0001).

Many reports have demonstrated that the cytoprotective roles of FOXOs are mainly attributable to antioxidative function in response to oxidative stress (35). Therefore, we examined whether 6-OHDA-induced FOXO4 degradation affected cell viability and whether FBXO7 played a role during the process. Firstly, we checked the transfection efficiency of *FBXO7*-siRNA and *FOXO4*-siRNA in MN9D cells. Immunoblotting analysis revealed that the expression of endogenous FBXO7 and FOXO4 was efficiently downregulated by its siRNA. In addition, endogenous FOXO4 level was correspondingly increased by *FBXO7*-siRNA (Fig. 7, *A* and *B*).

After MN9D cells were treated with various concentrations of 6-OHDA, cell viability was measured using LDH cytotoxicity assays. As shown in Figure 7*C*, MN9D cell cytotoxicity increased in the presence of 6-OHDA in a dose-dependent manner. MN9D cells transfected for 48 h with control siRNA, siRNA-*FOXO4*, or siRNA-*FBXO7* were treated with 100 μM 6-OHDA or vehicle. Cells that underwent siRNA-mediated *FOXO4*-knockdown alone exhibited a greater than 1.7-fold increase in 6-OHDA-induced cytotoxicity compared with that of cells transfected with control siRNA (Fig. 7*D*). Meanwhile, cells that underwent siRNA-mediated *FBXO7*-knockdown alone showed approximately 7% lower levels of 6-OHDA-induced cytotoxicity compared with that of cells transfected with control siRNA (Fig. 7*D*). When cells were cotransfected with both *FOXO4*-siRNA and *FBXO7*-siRNA and then treated with 6-OHDA, cellular cytotoxicity decreased by more than 2-fold compared with that of cells that underwent *FOXO4*-knockdown alone (Fig. 7*D*). Taken together, these findings suggested FBXO7 potentiated the cytotoxicity of 6-OHDA in neuronal cells and that this occurred through the degradation FOXO4 and consequent blockade of the cytoprotective effect of FOXO4.

### The PD-linked FBXO7-T22M mutant reduced FOXO4 stability much greater than wild-type FBXO7

**Figure 8:**
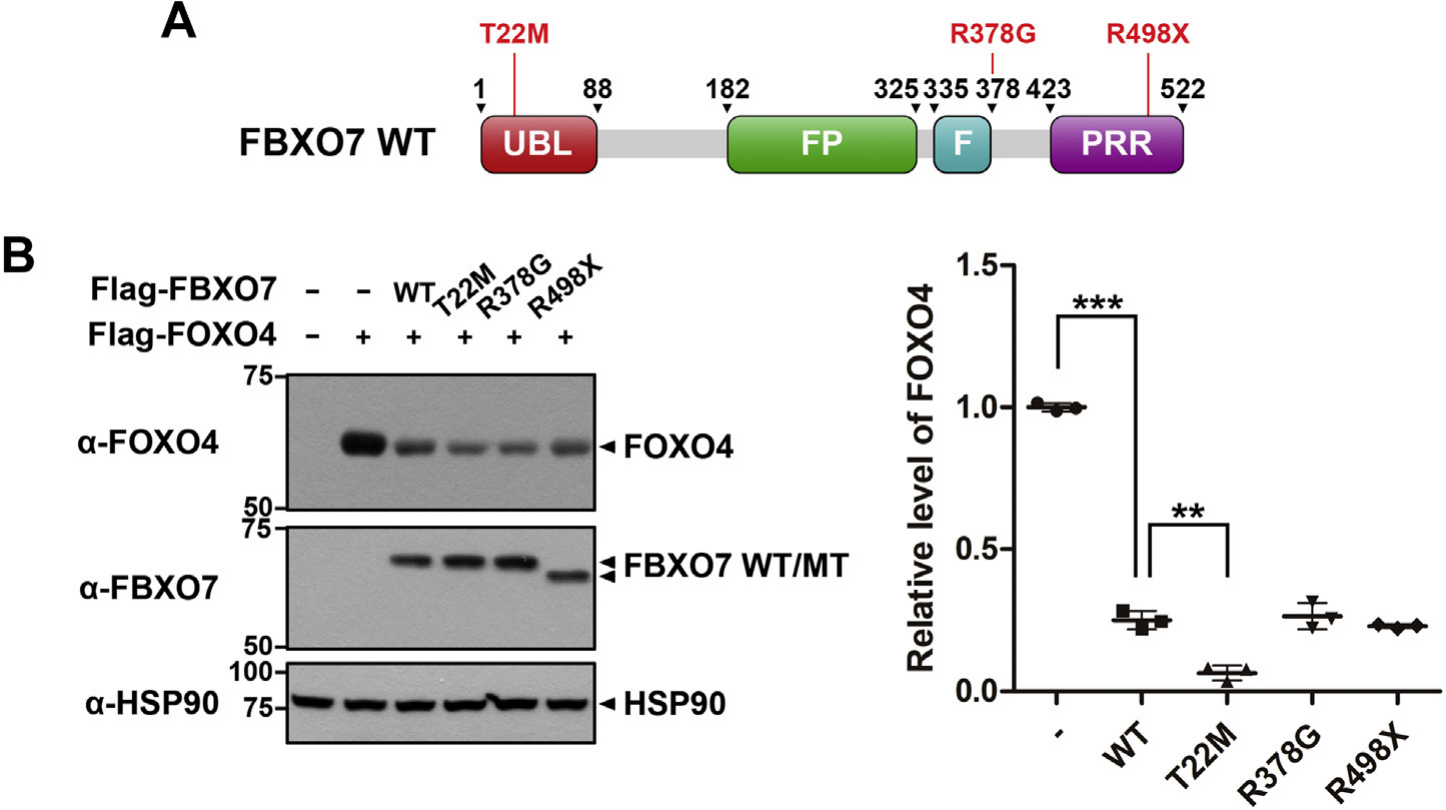
**The PD-linked FBXO7-T22M mutant reduces FOXO4 stability much greater than wild-type FBXO7.***A*, Schematic of PD-linked mutations in FBXO7. *B*, HEK293 cells were transfected for 24 h with plasmids encoding FLAG-FOXO4, wild-type FLAG-FBXO7, mutant FLAG-FBXO7-T22M, mutant FLAG-FBXO7-R378G, or mutant FLAG-FBXO7-R498X, either alone or in combination. Cell lysates were immunoblotted and screened using the indicated antibodies. Relative levels of FOXO4 were quantified and the results presented as the mean ± SD of three independent experiments (∗∗∗*p* ≤ 0.0001; ∗∗*p* ≤ 0.001). Hsp90 served as a loading control.

We then investigated the effect of familial PD-linked pathogenic mutations of FBXO7 on FOXO4 degradation. Four missense or nonsense point mutations have been reported to date in *FBXO7* that are linked to familial PD (36). We selected three of the mutations (T22M, R378G, and R498X) to examine their effect on FOXO4 degradation. The T22M mutation is located in the N-terminal Ubl domain, the R378G mutation is located at the end of the F-box domain, and the R498X mutation is in the C-terminal proline-rich region (PRR) (Fig. 8*A*). Cells were transfected with *FOXO4*, either alone or in combination with wild-type *FBXO7* or one of the *FBXO7* mutants. All three point mutants of *FBXO7* reduced the levels of FOXO4, similar to the pattern observed with the transfection of wild-type *FBXO7*. In particular, the *FBXO7*-T22M mutant facilitated the degradation of FOXO4 approximately 3.9-fold greater than that of wild-type *FBXO7* (Fig. 8*B*). These results indicated that the *FBXO7* T22M mutation within the Ubl domain may affect the proteolytic activity of FBXO7, possibly by increasing FBXO7-binding to FOXO4 or by further stimulation of the novel caspase 8-linked pathway. This could consequently contribute to more severe dysfunction of FOXO4 in variant FBXO7-linked familial PD.

## Discussion

Age-related changes in a number of cellular processes, such as excessive oxidative stress and mitochondrial dysfunction, can promote cell death, including neuronal cell death (37, 38, 39, 40). Accordingly, aging is a major risk factor for the development of NDDs. FOXO transcription factors are important determinants in extending the human life span, and their functional defects are consequently involved in age-related diseases, such as cancer, diabetes, and NDD. Similar to other FOXOs, FOXO4 is associated with longevity through the insulin/IGF-1 signaling pathway (15, 41). As many FOXOs are essential for neuronal survival or apoptosis (19), FOXO4 may be linked to neuronal cell death and PD pathogenesis; however, its molecular mechanism has not been elucidated. Here we found that PD-associated FBXO7 promoted the degradation of FOXO4 in mammalian cells. Moreover, the cytoprotective levels of FOXO4 were greatly reduced in dopaminergic MN9D cells treated with 6-OHDA through the action of FBXO7 and caspase 8.

According to multiple studies, the cytoprotective effect of FOXO4 is attributable to its antioxidant activity. For example, FOXO4 promotes the detoxification reaction through transcriptional activation of antioxidant enzymes, such as superoxide dismutase 2 and catalase (42). In contrast to FOXO4, FBXO7 is able to exert either cytoprotective or cytotoxic action in response to stress, depending on the differential cellular context. Regarding the neurotoxic effect, FBXO7 promotes PINK1 ubiquitination and degradation. The FBXO7 small-molecule inhibitor increases PINK1 stability by reducing the interaction between FBXO7 and PINK1. As PINK1 has cytoprotective effects, the treatment of FBXO7 inhibitors improves cell viability and reduces dendrite injury against 1-methyl-4-phenylpyridium ion (MPP+) toxicity in human SH-SY5Y cells and primary neurons (43). On the other hand, soluble wild-type FBXO7 exhibits cytoprotective activity. For example, mild and transient stress can induce FBXO7 expression to protect cells, which facilitates neuroprotective mitophagy (44). The current study provided additional evidence that FBXO7 displays neurotoxic action, which may be produced in part *via* FOXO4 degradation. Furthermore, it was found that FBXO7 exaggerated MN9D cell death in response to 6-OHDA. Interestingly, all three PD-linked FBXO7 mutants reduced FOXO4 degradation, wherein the FBXO7-T22M mutant’s effect was most pronounced.

In regard to the SCF-independent noncanonical function of FBXO7, FBXO7 affects the biochemical and functional activity of its multiple binding partners. For example, binding of FBXO7 to Cdk6 does not change its expression levels, but does enhance Cdk6 activity, suggesting that FBXO7 acts as an assembly scaffold for the formation of the cyclin D/Cdk6 complex (12). The current study suggested that FBXO7 also interacted with caspase 8, triggering its activation and leading to FOXO4 degradation.

In eukaryotic cells, intracellular protein degradation is mediated by two major pathways, the ubiquitin-proteasome pathway and lysosomal autophagy pathway (24). In the current study, we attempted to clarify which of these systems was involved in FOXO4 degradation. We showed that a caspase inhibitor markedly blocked the FBXO7-mediated proteolysis of FOXO4, but this effect was not observed with proteasome or lysosome inhibitors. These results demonstrated that FBXO7 reduced the protein stability of FOXO4 through an SCF-independent caspase-linked pathway. Consistent with our findings, a previous study reported that treatment with a proteasome inhibitor fails to rescue Mdm2-mediated FOXO4 degradation (45). In support of our finding, FOXO4 is reported to be a target of degradation *via* both the non-UPS and UPS pathways. Concerning FOXO4 degradation through non-UPS pathway, Mdm2 acts as an E3 ligase for FOXO4 degradation and mediates its mono-ubiquitination. Mdm2-mediated reduction of FOXO4 levels is not rescued by MG132 treatment (45). In contrast, the viral oncoprotein Tax induces FOXO4 degradation and generates its cleavage products *via* the proteasome pathway in human T-cell leukemia virus type 1 (HTLV-1)-infected cells (46). Moreover, in RAS-mutant cancer cells, casein kinase 1α phosphorylates FOXO4 at Ser265 and Ser268, leading to its proteolysis by nuclear proteasomes (47).

We also demonstrated that caspase 8 binds to both FBXO7 and FOXO4. Although protease-dependent protein degradation is a minor pathway, several proteins have been identified as targets of the caspase-dependent pathway (48, 49). No known target has been reported as substrate(s) of caspase 8, except for cIAP-1. As a substrate for caspase 8, cIAP-1 is likely to have multiple cleavage sites. TNF-related apoptosis-inducing ligand (TRAIL)-induced apoptosis of liver cancer cells is associated with the degradation of cIAP-1 and X-linked IAP (XIAP) (50). Pan-caspase inhibitor Q-VD-OPH stabilizes cIAP-1 protein levels during TRAIL treatment, but lysosomal inhibitors (Bafilomycin A1, CRA025850) and proteasome inhibitor (MG132) do not. Specifically, *caspase-8* knockdown stabilizes both cIAP-1 and XIAP, while *caspase-9* knockdown prevents XIAP degradation, but not cIAP-1 degradation (50). These results suggest that TRAIL-induced cIAP-1 degradation requires caspase-8 activity. While our current data suggested that FBXO7-mediated caspase 8 activation appeared to be indirectly involved in the proteolysis of FOXO4, further study is necessary to clarify the entire pathway and to characterize the unknown target(s) participating in the degradation of FOXO4 after caspase 8 activation.

Mutation of *FBXO7* has been found to cause autosomal recessive early-onset PD (4). For example, the R378G mutation of *FBXO7* has been shown to reduce the affinity of the SCF complex for Skp1, whereas the R498X mutation results in the removal of 24-amino acids from the substrate-binding domain, reducing SCF-dependent E3 ligase activity. In addition, the T22M mutation in the Ubl domain interferes with its interaction with parkin, preventing its translocation to depolarized mitochondria and consequently suppressing mitophagy (51, 52, 53). Interestingly, all three pathogenic PD-linked point mutants of FBXO7 reduced the degradation of FOXO4 in our current study with the strongest effect being observed for the FBXO7-T22M mutant.

Both 6-OHDA and MPP+ are neurotoxins commonly used to induce PD symptoms in mice. A previous study revealed that cytochrome *c* is released into the cytosol in dopaminergic neurons and MN9D dopaminergic cells after 6-OHDA and MPP+ treatment, respectively. Meanwhile, treatment with a caspase inhibitor blocks 6-OHDA-mediated neurotoxicity, but not MPP+ cytotoxicity (54, 55). These results indicate that 6-OHDA treatment induces caspase-dependent neuronal cell death. This hypothesis was further supported by our current finding that 6-OHDA-induced degradation of FOXO4 in MN9D cells occurred through the FBXO7-mediated novel caspase-linked pathway, specifically, *via* caspase 8.

In conclusion, based on findings of the present study, we propose a new regulatory pathway for the action of FOXO4. The findings also suggest that FBXO7-mediated proteolysis of FOXO4 may contribute to the pathogenesis of PD through the cytotoxic effect of FBXO7.

## Experimental procedures

### Materials

Dulbecco’s modified Eagle’s medium (DMEM), fetal bovine serum (FBS), Lipofectamine 2000, polyethylenimine (PEI) reagent, and anti-rabbit and anti-mouse horseradish peroxidase (HRP)-conjugated secondary antibodies were purchased from PerkinElmer Life and Analytical Sciences. Protein A Sepharose beads were purchased from GE Healthcare Biosciences. Proteasome inhibitor MG132 was purchased from A.G. Scientific, and epoxomicin was purchased from Millipore. NH_4_Cl and cycloheximide were purchased from Sigma-Aldrich. PI3K inhibitor LY294002 and caspase inhibitor Z-VAD-FMK were purchased from Calbiochem, and caspase-8 inhibitor Z-IETD-FMK was purchased from Santa Cruz Biotechnology. Enhanced chemiluminescence (ECL) reagent was purchased from Abclon and Advansta. All other chemicals used in the study were analytical-grade commercial products and purchased from Sigma-Aldrich. Primary antibodies for western blotting included mouse monoclonal anti-Myc (1:1000, sc-40, Santa Cruz Biotechnology), anti-FLAG (1:1000, F3165-1MG, Sigma-Aldrich), anti-HA (1:1000, sc-7392, Santa Cruz Biotechnology), anti-V5 (1:10,000, R960-25, Invitrogen), anti-FBXO7 (1:1000, sc-271763, Santa Cruz Biotechnology), anti-caspase 8 (1:1000, 9746S, Cell Signaling Technology), anti-tubulin (1:10,000, sc-8017, Santa Cruz Biotechnology), anti-Hsp90 (1:10,000, sc-13119, Santa Cruz Biotechnology), anti-actin (1:10,000, sc-47778, Santa Cruz Biotechnology), and anti-GAPDH (1:10,000, sc-32233, Santa Cruz Biotechnology) and rabbit monoclonal anti-FOXO4 (1:5000, ab128908, Abcam), anti-FBXO7 (1:1000, sc-86450, Santa Cruz Biotechnology), and anti-p27kip1 (1:500, #2552, Cell Signaling Technology). Secondary antibodies were HRP-conjugated anti-mouse (AP124P) and anti-rabbit (AP132P) purchased from EMD Millipore.

### DNA constructs and RNA interference

Mammalian expression vectors for FLAG-tagged human wild-type FBXO7 isoform-1 and its deletion mutants were provided by Dr H. J. Kuiken (The Netherlands Cancer Institute, Amsterdam, Netherlands). Mammalian expression vectors for all caspase constructs were provided by Dr Y. K. Jung (Seoul National University, Seoul, Republic of Korea). The mammalian expression vector for FLAG-tagged human FOXO4 (plasmid #17549) and pGL3-p27Luc vector (plasmid #23047) were purchased from Addgene Korea. All plasmid sequences were verified by DNA sequencing (COSMO Genetech). Small interfering RNAs (siRNAs) for FBXO7 and FOXO4 and control scrambled siRNA (cat. # 51-01-14-04) were designed and synthesized by Integrated DNA Technologies (Hanam-si). The *FBXO7*-specific siRNA duplex sense and antisense sequences were 5′-UUGGUUCUCCUCUAGAUUGAAGUdTdT-3′ and 5′-ACUUCAAUCUAGAGGAGAACCAAdTdT-3′, respectively. The *FOXO4*-specific siRNA duplex sense and antisense sequences were 5′-UGAUAGUGACAUGAUACAAACACdTdT-3′ and 5′-GUGUUUGUAUCAUGUCACUAUCAdTdT-3′, respectively. The *caspase 8*-specific siRNA was designed and synthesized by Bioneer. The *caspase 8*-specific siRNA duplex sense and antisense sequences were 5′-GCUGCUCUUCCGAAUUAAUdTdT-3′ and 5′-AUUAAUUCGGAAGAGCAGCdTdT-3′, respectively.

### Cell culture and DNA transfection

Human embryonic kidney 293 (HEK293) cells and human breast cancer MCF7 cells were maintained in DMEM supplemented with 10% FBS and 100 U/ml penicillin-streptomycin. *FBXO7*- knockout HAP1 cells (#HZGHC002684c003; Horizon Discovery) were maintained in Iscove's Modified Dulbecco's Medium supplemented with 10% FBS and 100 U/ml penicillin-streptomycin. Mouse neuroblastoma MN9D cells were cultured on dishes coated with 25 μg/ml Poly-D-lysine (Sigma-Aldrich) in high-glucose DMEM supplemented with 10% FBS and 100 U/ml penicillin-streptomycin in an atmosphere of 90% air and 10% CO_2_. Human neuroblastoma SH-SY5Y cells were maintained in DMEM/F12 supplemented with 10% FBS and 100 U/ml penicillin-streptomycin. All cells were grown at 37 °C with 5% CO_2_ unless indicated otherwise. All DNA transfections were carried out using Lipofectamine 2000 or PEI reagent according to the manufacturer’s instructions.

### Coimmunoprecipitation and immunoblot analysis

Cell lysates were washed with ice-cold phosphate-buffered saline (PBS), scraped, and mixed with 1% Nonidet P-40 lysis buffer (50 mM Tris, pH 7.5; 1% Nonidet P-40; 150 mM NaCl; 10% glycerol; 1 mM sodium orthovanadate; 1 μg/ml aprotinin; 1 mM EGTA; 1 mM sodium fluoride; and 0.2 mM phenylmethylsulfonyl fluoride). The cells were sonicated and the supernatants then collected by centrifugation at 13,000*g* for 15 min at 4 °C. For immunoprecipitation, 500–1000 μg of cell lysates was incubated with 1 μg of appropriate antibodies overnight at 4 °C with gentle rotation. Protein A Sepharose beads were then added and incubated for 2 h at 4 °C with gentle rotation. The beads were washed with 1% Nonidet P-40 lysis buffer and the immunocomplexes dissociated by boiling in 2× sample buffer. The samples were resolved by SDS-PAGE and transferred to a nitrocellulose membrane (Whatman, GE Healthcare Life Sciences). The membranes were blocked in TBST buffer (20 mM Tris, pH 7.5; 137 mM NaCl; and 0.1% Tween 20) plus 5% nonfat dry milk for 1 h at room temperature. The membranes were then incubated with the appropriate primary antibody at 4 °C overnight. Membranes were washed in TBST and incubated with the HPR-conjugated secondary antibody for 2 h at room temperature. The membranes were washed with TBST and the protein bands visualized using ECL reagents, following the manufacturer’s instructions.

### Preparation of mouse whole-brain lysates

Whole brains were obtained from 6-week-old male C57BL/6 mice (Orient). The brain tissues were homogenized and sonicated in lysis buffer containing 50 mM Tris (pH 7.4), 150 mM NaCl, 1% Triton X-100, 0.5% sodium deoxycholate, 0.1% SDS, and a protease inhibitor cocktail (Sigma-Aldrich). The samples were centrifuged at 13,000*g* for 20 min at 4 °C and the supernatants collected.

### Immunocytochemistry analysis

Human SH-SY5Y cells were seeded onto poly D-lysine-coated cover glasses. The adherent cells were washed twice with PBS and immediately fixed in 3.7% formaldehyde for 10 min at room temperature. After fixation, the cells were permeabilized with 0.1% Triton X-100 for 10 min and blocked with 1% BSA in TBST for 1 h at room temperature. The cells were immunostained using rabbit polyclonal anti-FOXO4 and/or mouse monoclonal anti-FBXO7 antibodies, washed, and incubated with Alexa Fluor 488- or Alexa Fluor 594-conjugated anti-IgG antibodies. Images were captured using an LSM 880 confocal microscope (Carl Zeiss) and processed using a Zeiss LSM Image Browser (Carl Zeiss).

### RNA extraction and real-time analysis

Total RNA was isolated from cells and target mRNAs were amplified by real-time PCR using complementary DNA (cDNA) as the template. The primer sequences for the *p27kip1* and GAPDH genes were as follows: p27kip1-forward, 5′-AAGGGCCAACAGAACAGAAC-3′; p27kip1-reverse, 5′-GGATGTCCATTCAATGGAGTC-3′, GAPDH-forward, 5′-TGCACCACCAACTGCTTAGC-3′, and GAPDH-reverse, 5′-GGCATGGACTGTGGTCATGAG-3′.

### Luciferase reporter assays

MCF7 cells were transfected with the luciferase reporter plasmid pGL3-p27-Luc. The cells were cotransfected with pRL-Luc vector, which was used to normalize the transfection efficiency. After 24 h of DNA transfection, cell lysates were collected and firefly and Renilla luciferase activities were measured using the Dual-Luciferase Reporter Assay System (Promega), following the manufacturer’s instructions. All firefly luciferase measurements were normalized to the Renilla luciferase measurements from the same samples.

### Lactate dehydrogenase (LDH) cytotoxicity assays

Cytotoxicity was evaluated using an LDH Cytotoxic Detection Kit (Takara). After siRNA transfection for 48 h, MN9D cells were treated with 100 μM 6-OHDA for an additional 6 h. Cell-free culture media was then collected and used in the LDH assay, according to the manufacturer’s instructions. The maximum LDH release (referred to as “high control”) was determined by solubilizing cells in 1% Triton X-100; the spontaneous LDH release (referred as “low control”) was determined by incubating cells in medium alone. Absorbance was measured at 490 nm using a microplate reader. The percentage of cytotoxicity was determined using the formula: Cytotoxicity = [(experimental value – low control)/(high control – low control)] × 100%.

### Statistical analysis

Statistical analysis of data from different groups was performed using an unpaired Student’s *t* test. All graphs were generated using GraphPad Prism 5.0 program (GraphPad Software, Inc). All values are reported as mean ± Standard Deviation (SD). The intensities of the western blot bands were measured using GelQuant.NET software (version 1.8.2).

## Data availability

All data that support the findings of this study are contained within the manuscript and are available from the corresponding author upon reasonable request.

## Supporting information

This article contains supporting information.

## Conflict of interest

The authors declare that they have no conflict of interest with the content of this article.
