# Supplementary material for: FBXO7 triggers caspase 8-mediated proteolysis of the transcription factor FOXO4 and exacerbates neuronal cytotoxicity
Source: J Biol Chem. 2021 Nov 17;297(6):101426. doi: 10.1016/j.jbc.2021.101426 (PMC8665361; doi:10.1016/j.jbc.2021.101426)
Supplement: Figures S1–S4 [file mmc1.pdf]

Supporting information

FBXO7 triggers FOXO4 proteolysis and exacerbates neuronal cytotoxicity

Su Hyoun Lee, Sungyeon Jung, Yun Ju Lee, Minju Hyun, and Kwang Chul Chung

Content: Supporting Figures and Legends

1. Figure S1
2. Figure S2
3. Figure S3
4. Figure S4

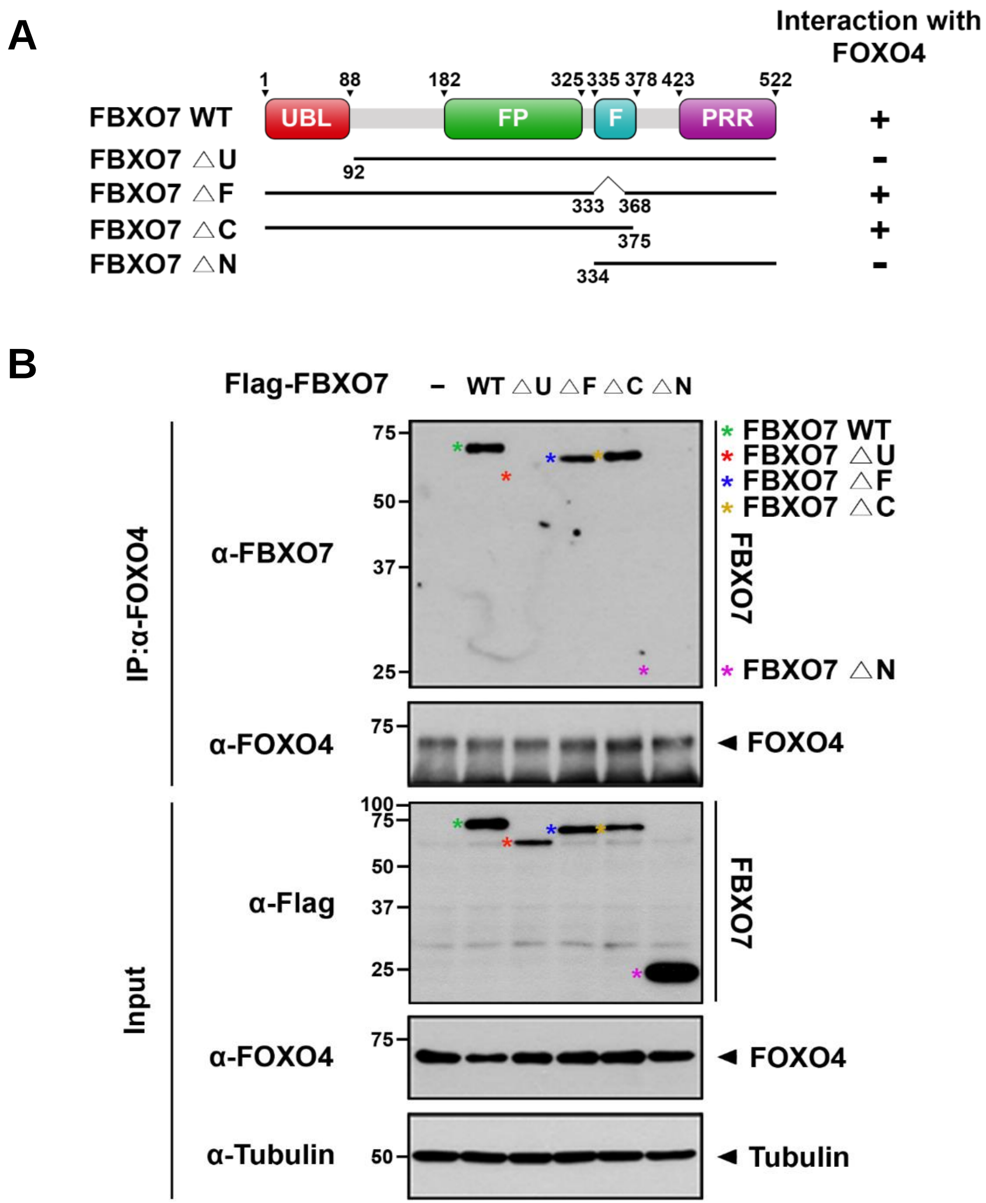

A
Interaction with FOXO4
1
88
182
325
335
378
423
522
FBXO7 WT
UBL
FP
F
PRR
+
FBXO7 △U
92
-
FBXO7 △F
333
368
+
FBXO7 △C
375
+
FBXO7 △N
334
-
B
Flag-FBXO7
–
WT
△U
△F
△C
△N
IP:α-FOXO4
α-FBXO7
75
50
37
25
FBXO7 WT
FBXO7 △U
FBXO7 △F
FBXO7 △C
FBXO7
FBXO7 △N
α-FOXO4
75
FOXO4
Input
α-Flag
100
75
50
37
25
FBXO7
α-FOXO4
75
FOXO4
α-Tubulin
50
Tubulin

Figure S1. The Ubl domain of FBXO7 is important for binding to FOXO4. *A*, Schematic of wild-type FBXO7 (FBXO7-WT) and its deletion mutants. Binding assay results of FBXO7 and its deletion mutants to FOXO4 are shown on the right side. Minus (−) indicates no binding and plus (+) indicates binding. *B*, HEK293 cells were transfected for 24 h with plasmids encoding FLAG-tagged wild-type FBXO7 or its deletion mutants and then treated for additional 6 h with 10 μM MG132. Cell lysates were immunoprecipitated using anti-FOXO4 antibody, followed by immunoblotting with the indicated antibodies. Tubulin served as a loading control.

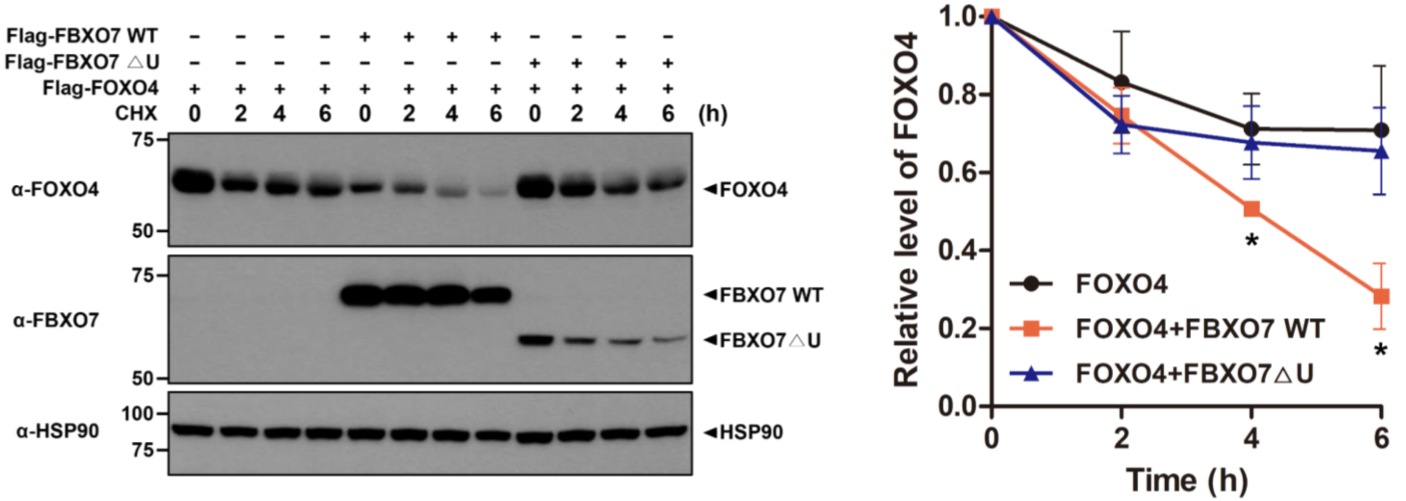

Figure S2. Wild-type FBXO7, but not FBXO7-ΔU mutant, decreases the protein stability of FOXO4. HEK293 cells were transfected for 24 h with plasmids encoding FLAG-FOXO4, either alone or in combination with a plasmid encoding FLAG-FBXO7-WT or FLAG-FBXO7-ΔU. Cells were then treated with 25 µg/ml cycloheximide for the indicated times and cell lysates were immunoblotted with the indicated antibodies. Relative levels of FOXO4 were quantified and the results presented as the mean ± SD of three independent experiments (*, $p \leq 0.05$). HSP90 served as loading controls.

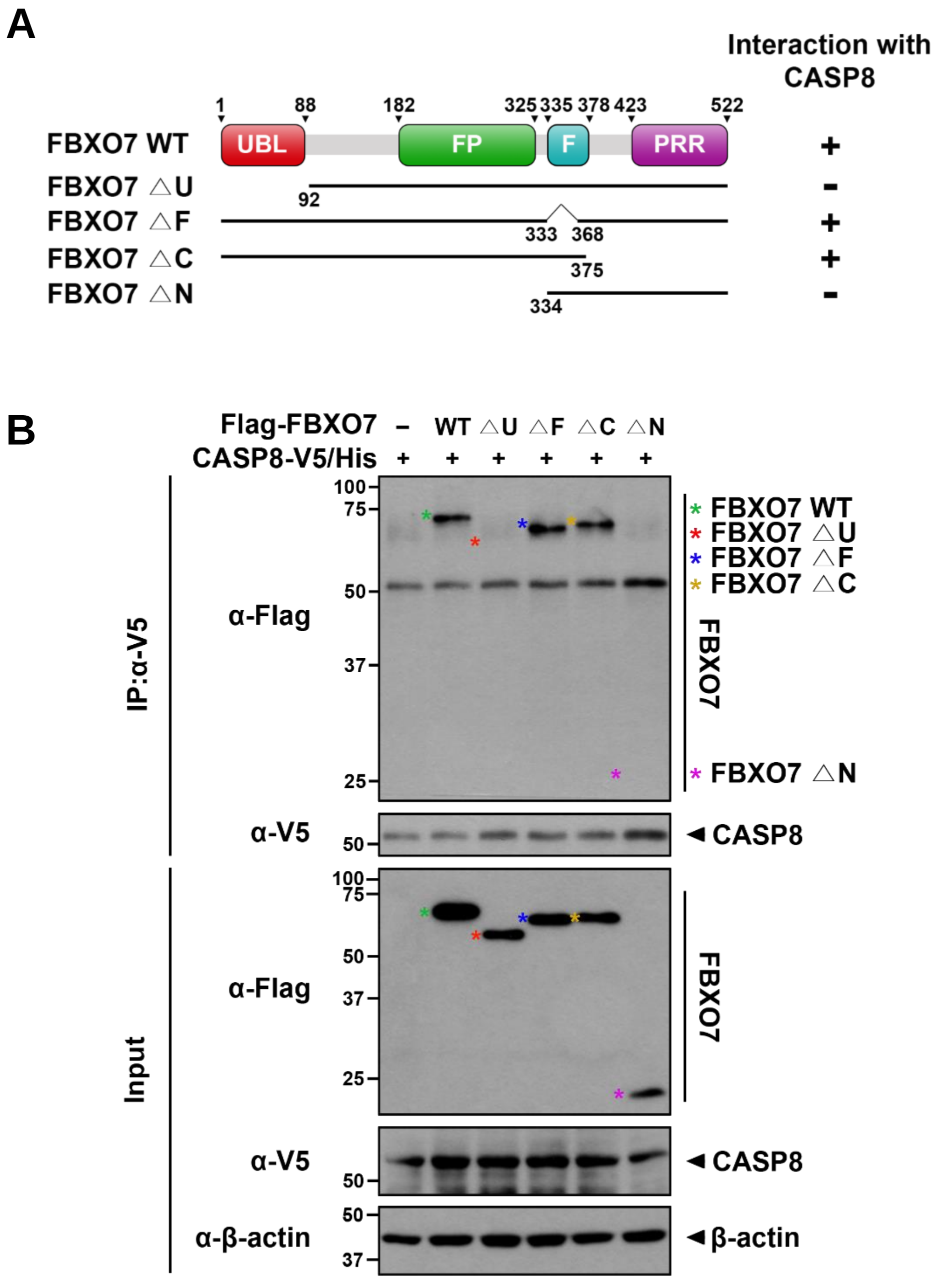
A
Interaction with CASP8
1
88
182
325
335
378
423
522
FBXO7 WT
UBL
FP
F
PRR
+
FBXO7 △U
92
-
FBXO7 △F
333
368
+
FBXO7 △C
375
+
FBXO7 △N
334
-
B
Flag-FBXO7
–
WT
△U
△F
△C
△N
CASP8-V5/His
+
IP:α-V5
α-Flag
100
75
50
37
25
FBXO7 WT
FBXO7 △U
FBXO7 △F
FBXO7 △C
FBXO7
FBXO7 △N
α-V5
CASP8
Input
α-Flag
FBXO7
α-V5
CASP8
α-β-actin
β-actin

Figure S3. The Ubl domain of FBXO7 is required for the interaction with caspase 8 . *A*, Schematic of wild-type FBXO7 (FBXO7-WT) and its deletion mutants. Binding assay results of FBXO7-WT and its deletion mutants to the caspase 8 to are shown on the right side. Minus (−) indicates no binding and plus (+) indicates binding. *B*, HEK293 cells were transfected for 24 h with the plasmid encoding caspase 8-V5/His, either alone or in combination with plasmid encoding FLAG-tagged FBXO7-WT or its deletion mutants. Cell lysates were immunoprecipitated using anti-FOXO4 antibody, followed by immunoblotting with the indicated antibodies. β-Actin served as a loading control.

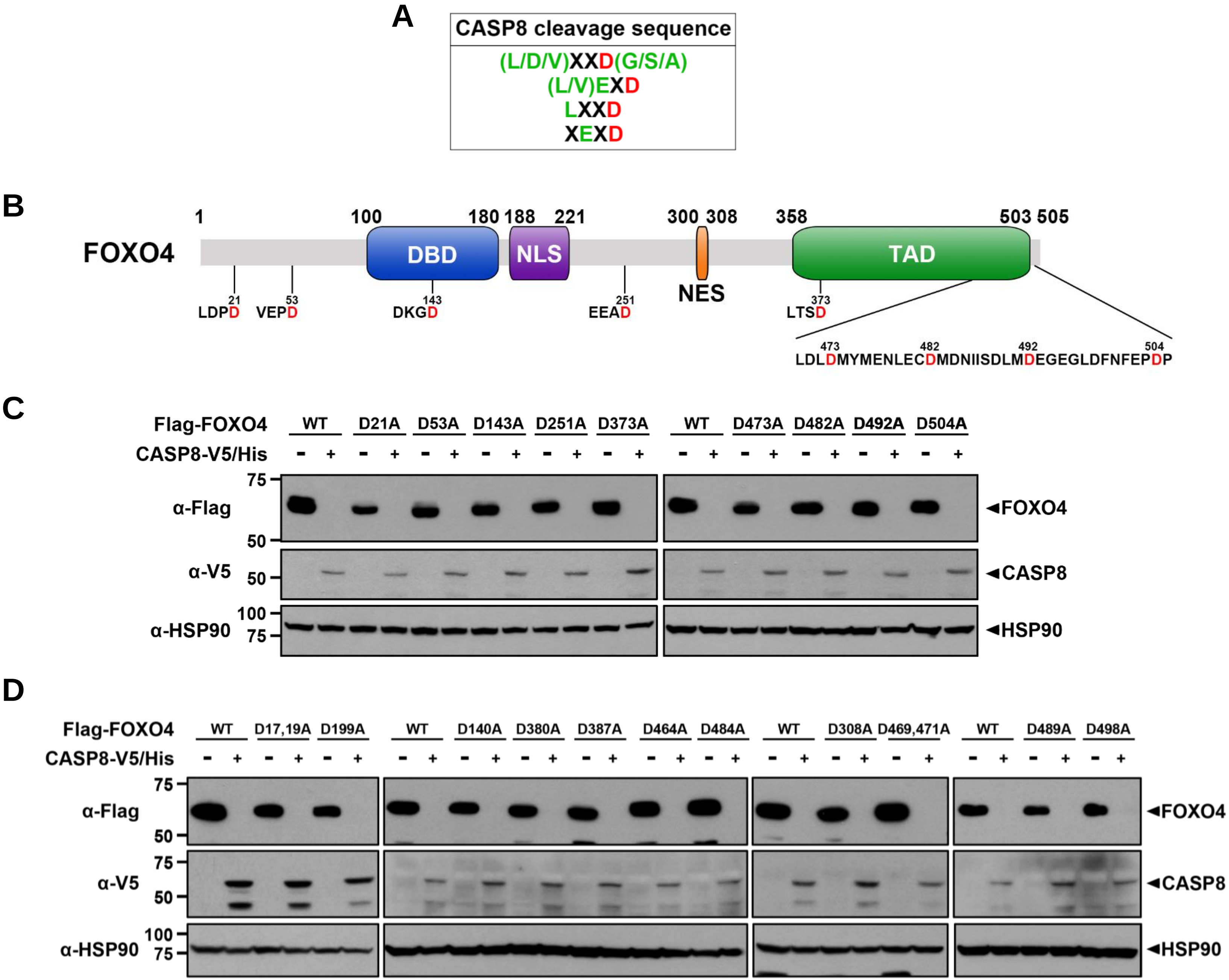
A
CASP8 cleavage sequence
(L/D/V)XXD(G/S/A)
(L/V)EXD
LXXD
XEXD
B
FOXO4
1
100
180 188
221
300 308
358
503 505
DBD
NLS
NES
TAD
21 LDPD
53 VEPD
143 DKGD
251 EEAD
373 LTSD
473 482 492 504
LDLDMYMENLECDMDNIISDLMDEGEGLDFNFEPDP
C
Flag-FOXO4
WT D21A D53A D143A D251A D373A WT D473A D482A D492A D504A
CASP8-V5/His
α-Flag
α-V5
α-HSP90
75
50
100
◄FOXO4
◄CASP8
◄HSP90
D
Flag-FOXO4
WT D17,19A D199A WT D140A D380A D387A D464A D484A WT D308A D469,471A WT D489A D498A
CASP8-V5/His
α-Flag
α-V5
α-HSP90
75
50
100
◄FOXO4
◄CASP8
◄HSP90

Figure S4. Mapping the cleavage site(s) of caspase 8 within the FOXO4. *A*, The four preferred amino acid sequences including cleaved Asp by caspase 8 are denoted. *B*, Schematic diagram of 9 Asp residues to be a target of point-mutation in human FOXO4 protein. Based on the sequences in (A), these sites were highly predicted to be the cleavage site(s) by caspase 8, and were replaced with Ala, respectively. *C*, Where specified, HEK293 cells were transfected for 24 h with plasmid encoding FLAG-FOXO4-WT, one of its 9 point-mutants, and/or caspase 8-V5/His and cell lysates were immunoblotted with the indicated antibodies. D, HEK293 cells were transfected for 24 h with the plasmids encoding FLAG-FOXO4-WT, one of its 13 point-mutants having the substation at the indicated single or double Asp sites, and/or caspase 8-V5/His and cell lysates were immunoblotted with the indicated antibodies. HSP90 served as loading controls.
